# Quantitative analysis of venous outflow with photo-plethysmography in patients with suspected thoracic outlet syndrome

**DOI:** 10.3389/fcvm.2022.803919

**Published:** 2022-10-28

**Authors:** Jeanne Hersant, Pierre Ramondou, Charlotte Josse, Simon Lecoq, Samir Henni, Pierre Abraham

**Affiliations:** ^1^Vascular Medicine, University Hospital, Angers, France; ^2^UMR CNRS 1083 INSERM 6015, LUNAM University, Angers, France; ^3^Sports and Exercise Medicine, University Hospital, Angers, France

**Keywords:** thoracic outlet syndrome (TOS), photo-plethysmography (PPG), pathophysiology, venous outflow impairment, upper limb, arm abduction, venous volume

## Abstract

**Background:**

Venous compression is the second most frequent form of thoracic outlet syndrome (TOS). Although venous photo-plethysmography (PPG) has been largely used to estimate the consequences of chronic thromboses (Paget Schroetter syndrome), systematic direct quantitative recording of hemodynamic consequences of positional venous outflow impairment in patients with suspected TOS has never been reported.

**Objective:**

We hypothesized that moving the arms forward (prayer: “Pra” position) while keeping the hands elevated after a surrender/candlestick position (Ca) would allow quantification of 100% upper limb venous emptying (PPGmax) and quantitative evaluation of the emptying observed at the end of the preceding abduction period (End-Ca-PPG), expressed in %PPGmax.

**Materials and methods:**

We measured V-PPG in 424 patients referred for suspected TOS (age 40.9 years old, 68.3% females) and retrieved the results of ultrasound investigation at the venous level. We used receiver operating characteristics curves (ROC) to determine the optimal V-PPG values to be used to predict the presence of a venous compression on ultrasound imaging. Results are reported as a median (25/75 centiles). Statistical significance was based on a two-tailed *p* < 0.05.

**Results:**

An End-Ca-PPG value of 87% PPGmax at the end of the “Ca” period is the optimal point to detect an ultrasound-confirmed positional venous compression (area under ROC: 0.589 ± 0.024; *p* < 0.001). This threshold results in 60.9% sensitivity, 47.6% specificity, 27.3% positive predictive value, 79.0% negative predictive value, and 50.8% overall accuracy.

**Conclusion:**

V-PPG is not aimed at detecting the presence of a venous compression due to collateral veins potentially normalizing outflow despite subclavicular vein compression during abduction, but we believe that it could be used to strengthen the responsibility of venous compression in upper limb symptoms in TOS-suspected patients, with the possibility of non-invasive, bilateral, recordable measurements of forearm volume that become quantitative with the Ca-Pra maneuver.

**Clinical trial registration:**

[ClinicalTrials.gov], identifier [NCT04376177].

## Introduction

Due to the conflicts between the neurovascular bundle and osteo-tendineous and muscular structures, compression of the subclavian vein can result in outflow impairment during 90° upper limb abduction. Most subjects with positional neurovascular compression during abduction remain asymptomatic. When symptomatic, this positional neurovascular compression is called thoracic outlet syndrome (TOS) (1–5). Most symptomatic TOS are considered to result from neural compression. Thrombotic complication of the positional compression of the subclavian vein (Paget Schroetter syndrome) is reported to be the second most frequent form of TOS (6–8). Beyond these thrombotic complications, many patients experience transient positional venous outflow impairment by subclavian vein compression that can occasionally be symptomatic (McCleery syndrome) (9), but that mainly remain asymptomatic. We recently proposed the use of venous photo-plethysmography (VPPG) as a way of measuring the forearm swelling that may result from positional venous compression during a 90° abduction test (10) and showed that fingertip V-PPG cannot replace forearm VPPG for estimating venous outflow (11). VPPG is of interest because it is easily performed, low-cost, observer-independent and it allows continuous and bilateral forearm volume recordings. Unfortunately, VPPG is a semi-quantitative technique (12); the amplitude of VPPG changes is not reliable from one patient to another or with changes in probe positions (13–15). Consequently, how can we know whether the forearm is completely emptied during upper limb abduction? We proposed the Ca-Pra maneuver in the sitting or standing position (a modified version of the Roos test) as a way of answering this puzzling question (10). For the Ca-Pra maneuver, following 90° abduction (as in the surrender/candlestick: “Ca” position) we asked the patient to move the elbows forward while keeping the hands elevated at the same level as in the “Ca” position. This second position mimics the prayer “Pra” attitude and opens the costo-clavicular angle. It is then expected that in normal subjects no emptying (VPPG increase) shall occur when moving from “Ca” to “Pra” and VPPG should remain almost stable. On the contrary, if a venous outflow occurs in “Ca,” moving the arm to “Pra” shall result in an increase in the VPPG signal (further venous emptying resulting from incomplete emptying in the “Ca” position). Then, by allowing the recording of the VPPG value observed during complete emptying in all patients (10), the Ca-Pra maneuver allows for a quantitative analysis of the otherwise semi-quantitative VPPG technique (12).

The degree of emptying (percentage of maximal VPPG signal: %PPGmax) that should be used to discriminate normal from abnormal outflow has never been reported. Our aim was to determine this value using the presence of a compression on ultrasound investigation as a reference.

## Materials and methods

### Experimental design

The SKIPA database is a prospective ethically approved cohort database of all patients referred to the department of vascular medicine at the University Hospital of Angers. Patients with suspected TOS have recordings of symptoms, age, sex, weight, height, systolic and diastolic arm pressure and ongoing pain killer treatments. Patients self-completed the “disability of the arm and shoulder” (DASH) 38-item questionnaire, as routine during each visit. The score was calculated if at least 90% of the answers to the first 30 questions were available. All patients underwent ultrasound investigations as well as an evaluation of forearm volume changes with VPPG in a random order as described below. All patients were aware that their medical record could be used for research purposes and that they could object to this use.

### Ethical standards

This study was performed in compliance with the principles outlined in the Declaration of Helsinki and validated by the Ethics Committee in Angers (reference: 2020/17 and accessible on Clinicaltrial.gov under reference NCT04376177). As an observation of our medical routine and in accordance with French law, no individual consent was required, but all the patients were fully informed that they could deny the use of their medical file for research purposes. Patients denying the use of their data, unable to understand the information for linguistic or cognitive reasons, and under 18 years of age were not included in the analysis. For the present study we selected adult patients that had ultrasound (US) and PPG results from database initiation in 2019 to 27/09/2021. We excluded the patients that did not have PPG recordings or for whom the ultrasound report did not provide specific information about the presence or absence of positional venous compression during the provocation tests.

### Ultrasound imaging

Ultrasound investigations were performed by trained operators blinded to the results of PPG recordings. A large series of maneuvers can be performed to detect TOS, and the operators have their own routine, but all the operators performed at least a Roos test. For the present study, we encoded ultrasound venous results as positive or negative on each side for the presence of a venous compression during positional maneuvers, regardless of which maneuver resulted in a compression (because it was rarely reported by the operators). A report of a normal investigation was considered negative for a venous compression. Inversely, investigations reporting only the presence of an arterial compression without information on the presence or absence of a venous compression were excluded from the analysis.

### Photo-plethysmography recordings

VPPG tests were performed blinded to the results of ultrasound imaging. Venous photo-plethysmography (VPPG) was measured on each forearm at a sample rate of 4 Hz, 2–3 cm distal from the elbow crest using the Vasolab 320^®^ device (ELCAT, Germany). The system allows for the bilateral recording of low-pass filtered plethysmographic signal, it records 2 s at rest, used as zero volume, while the arms are alongside the torso for at least 30 s (complete filling) and then automatically stops after 60 s of recordings. Increases from zero denotes forearm emptying and vice versa. Values are recorded in arbitrary units (AU).

### Photo-plethysmography attitudinal maneuvers

**FIGURE 1:**
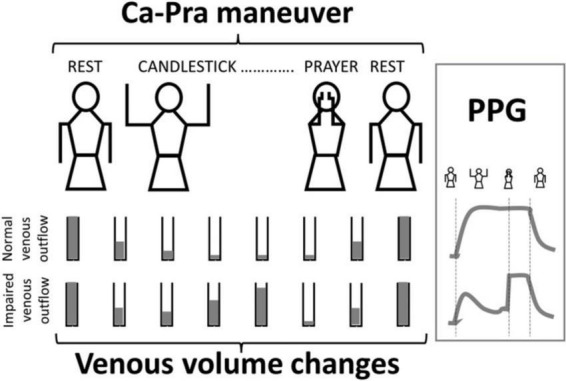
Schematic representation of venous volume changes and result of V-PPG changes in case of normal or impaired outflow during the Candlestick-Prayer maneuver.

We used the “Ca-Pra” maneuver, a slightly modified Roos test, during which after arm elevation, the candlestick/surrender (“Ca”) attitude is maintained until second 30 without opening and closing of the hands to prevent movement artifacts on PPG recordings. At second 30, a change to the prayer (“Pra”) position (arm elevation with elbows in front of the patient) was made and maintained until second 45. In the “Pra” position elbow and hands are at the same level relative to heart level as for the “Ca” position. The specific interest of the “Pra” position is to open the costo-clavicular angle and attain arm elevation without vascular compression. This procedure allows quantification of complete venous emptying (thus confirming the presence or absence of outflow impairment in the “Ca” position). If outflow was not impaired, forearm volume would remain unchanged between “Ca” and “Pra,” while it would decrease during “Pra” if venous outflow was impaired in the “Ca” position, as presented in Figure 1. At second 45 of the test, upper limbs were lowered.

### Data analysis

Values at forearm lowering were discarded from the recording because forearm lowering was not standardized. For the analysis, we recorded the maximal PPG observed during the Ca-Pra maneuver and the PPG value at the end of the “Ca” position (End-Ca-PPG) and we normalized End-Ca-PPG to highest PPG and expressed the results in %PPGmax.

### Statistical analysis

Kolmogorov–Smirnov tests were used to test the distribution of variables and results are presented as mean ± standard deviation (SD) for parametric, or median (25°/75°centiles) for non-parametric continuous variables. The receiver operating characteristics (ROC) curve analysis was performed to determine the performance of End-Ca-PPG [expressed in arbitrary units (AU) or as %PPGmax] and of PPGmax (in arbitrary units) to detect the presence of a compression at ultrasound. If significantly different from a random choice (area = 0.500), we searched for the thresholds to be used to detect the presence of a compression at ultrasound. From the ROC curve analysis, the distance of each point of sensitivity/specificity to the 100%/100% angle was calculated for each value of End-Ca-PPG. The End-Ca-PPG value that resulted in the shortest distance was determined because it is considered the optimal threshold to be used for an equal cost of false positive and false negative results. All statistical analyses were performed using SPSS (IBM SPSS statistics V15.0, Chicago, IL, USA). For all tests, a two-tailed *p* < 0.05 was considered to be statistically significant.

## Results

Over the study period, 485 patients were investigated for TOS. Ten were < 18 years and were not included in the database and US results were available for 442 of the other 475 patients. Of these patients, 51 did not have a PPG recording due to the ELCAT system being unavailable or due to technical failure on one or both sides. We then had 424 patients (848 upper limbs). A description of these patients is reported in Table 1.

**TABLE 1:** Description of the 424 studied patients.

Age (years)	40.9 ± 11.5 (min = 18; max = 82)
Males/females	130 (31.7%)/294 (68.3%)
Weight (kg)	72.0 ± 16.8 (min = 40; max = 149)
Height (cm)	167 ± 9 (min = 142; max = 196)
Pain killers	206 (50.6%)
… of which taken on a daily basis	111 (53.9%)
Rehabilitation	319 (77.8%)
… with improvement	111 (34.8%)
Pain by history	408 (96.2%)
… on the right side	127 (31.1%)
. on the left side	117 (28.7%)
… bilateral	164 (40.2%)
SF12 Physical Component Score	40.6 ± 11.1 (min = 19.6; max = 64.2)
SF12 Mental Component Score	38.2 ± 8.5 (min = 17.4; max = 59.6)
Systolic arm pressure (mmHg)	130 ± 17 (min = 100; max = 183)
Diastolic arm pressure (mmHg)	76 ± 12 (min = 46; max = 118)
Ultrasound positive right side	102 (25.1%)
Ultrasound positive left side	105 (25.8%)
End-Ca-PPG right side (AU)	6.0 (2.7/13.7) (min = −10; max = 31.7)
End-Ca-PPG left side (AU)	6.6 (3.1/15.3) (min = −3.2; max = 38.3)
Max PPG right side (AU)	9.0 (5.0/21.0) (min = 0.2; max = 32.1)
Max PPG left side (AU)	8.8 (5.2/21.2) (min = 0.7; max = 70.1)
%PPGmax right side	84 (46/97) (min = −575; max = 100)
%PPGmax left side	86 (56/98) (min = −257; max = 100)

**FIGURE 2:**
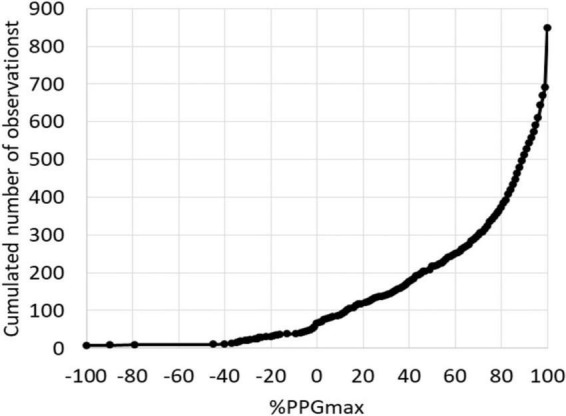
Distribution of the 848 values at the end of the candlestick period (End-Ca-PPG). Note that 6 values were < −100%PPGmax and 158 values were equal to 100%PPGmax.

As shown, patients were predominantly female. Half of the patients took painkillers on a regular basis. Sixteen of the patients did not report pain by history but were investigated for chronic forearm swelling, Raynaud syndrome or unexplained distal emboli. One hundred and forty-six (34.4%) patients had an ultrasound investigation reporting a venous compression on the right side (*n* = 41), left side (*n* = 44), or bilaterally (*n* = 61). As shown in Figure 2, absolute values for PPG at the end of the “Ca” period were extremely heterogeneous with 158 (18.6%) values being 100%PPGmax.

**FIGURE 3:**
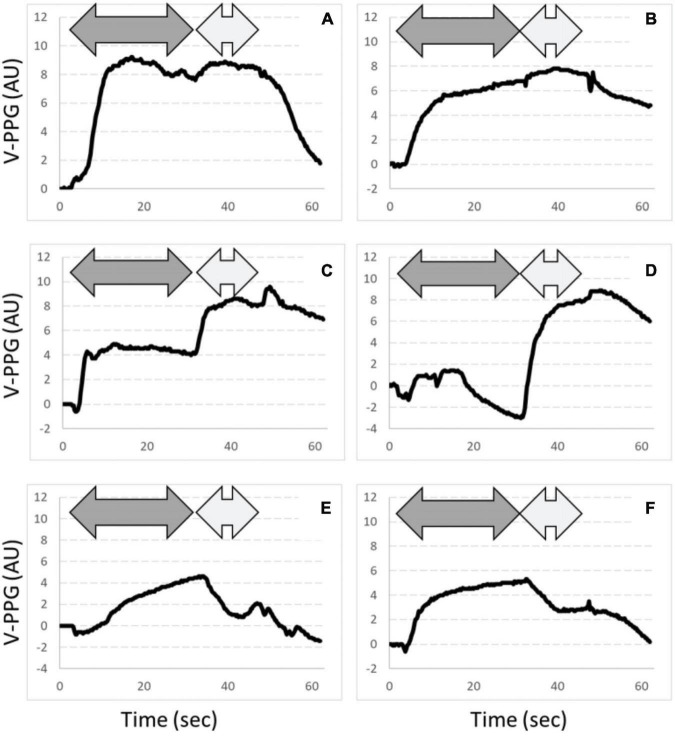
Examples of PPG changes observed. Dark gray arrows are “Ca” phases and light gray arrows are “Pra” phases of the Ca-Pra maneuver. Panels **(A,B)** are expected normal responses with complete emptying at the end of the “Ca” phase. Panels **(C,D)** show incomplete emptying during the “Ca” phase. Note that panel **(D)** shows a filling after the initial emptying, assumed to result from persistent inflow while outflow is impaired. In panels **(E,F)**, emptying reaches its maximal value at the end of the “Ca” phase but a venous filling occurs during the “Pra” phase. This filling is assumed to result from post-ischemic venodilatation due to inflow impairment during the “Ca” phase. Also note that for panel **(E)**, venous outflow is slowed, although reaching its maximum at the end of the “Ca” phase, suggesting that (if not resulting from a slow abduction) outflow was not abolished but severely impaired.

Typical examples of A-PPG recording in a patient with unilateral symptoms are presented in Figure 3. As shown, after an initial emptying, V-PPG absolute values could reach negative values during the “Ca” phase of the Ca-Pra maneuver (suggesting the presence of a persistent arterial inflow forcing blood into the vein while outflow is impaired) until elbow are moved forward and veins reach full emptying.

**FIGURE 4:**
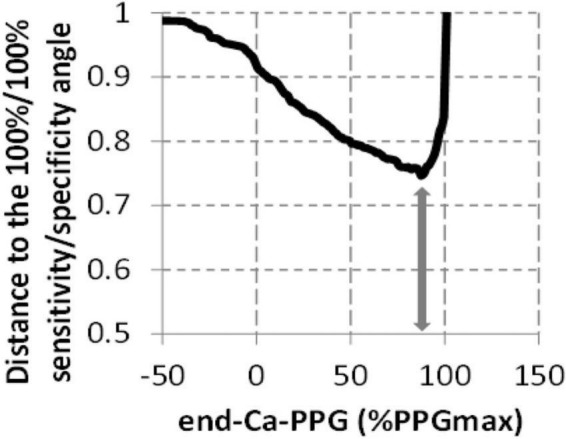
Determination of the threshold to be used for End-Ca-PPG (expressed as %PPGmax) to predict the presence of a venous compression on ultrasound imaging. The arrow represents the shortest distance to the angle and corresponds to a 87%PPGmax value.

Table 2 and Figure 4 show the result of ROC curve analysis on a limb-by-limb basis. As shown in Table 2, neither the absolute value of End-Ca-PPG nor PPGmax are significantly predictive of the presence of a venous compression at imaging. As shown, in Figure 4 the optimal cutoff point for End-Ca-PPG to predict the presence of a venous compression at ultrasound was 87%PPGmax. It is interesting to note that End-Ca-PPG values < 87%PPGmax were noted in 447 (52.7%) of the upper limbs. In 65 limbs (7.7% of the recordings) End-Ca-PPG was equal to or lower than zero, while in almost all cases PPG increased initially (forearm emptying) at the beginning of the candlestick period. This threshold results in 60.9% sensitivity, 47.6% specificity, 27.3% positive predictive value, 79.0% negative predictive value and 50.8% overall accuracy.

**TABLE 2:** Area for ROC curve analyses.

	Area of ROC	SD	*p*	Lower limit 95% CI	Upper limit 95% CI
End-Ca-PPG (AU)	0.527	0.024	0.250	0.479	0.574
PPGmax (AU)	0.457	0.023	0.063	0.411	0.503
End-Ca-PG (%PPGmax)	0.589	0.024	0.000	0.541	0.637

SD is standard deviation of the area.

## Discussion

This is the first study to report a quantitative analysis of VPP in patients with suspected TOS. As shown, an End-Ca-PPG of 87%PPGmax during the Ca-Pra maneuver is the optimal cut-off point to differentiate complete from incomplete upper limb emptying during 90° abduction. End-Ca-PPG values of < 87%PPGmax were found in 52.7% of recordings, suggesting that venous outflow impairment is very frequent in patients with suspected TOS.

Plethysmography is the generic name for various techniques that aim to measure the changes in the volume of tissue in humans (12, 13, 16). Among the available techniques are strain gauge plethysmography, conductance plethysmography, air plethysmography and photo-plethysmography (PPG) (17–22). Regardless of the technique used, there are generally two signals that can be extracted from the plethysmography signal. The first one corresponds to the small rhythmic changes resulting from arterial pulsatility and can be separated from the raw signal by a high-pass filter (23). The second one is a slow but ample component that mainly results from venous volume changes and can be obtained by applying a low-pass filter to the raw PPG signal (13, 24, 25). In patients with TOS, the high-pass filtered PPG signal (arterial pulsatility) has been largely studied (26–29), whereas low-pass filtered PPG (venous PPG) has scarcely been studied (10, 30). The specific interests of the Ca-Pra maneuver are to confirm whether venous emptying was impaired in abduction (“Ca” phase) and to change the semi-quantitative PPG technique (12) into a quantitative tool. In our previous report we showed that the V-PPG signal could be classified into 4 different patterns, two of which were assumed to result from venous outflow impairment and show venous emptying in the transition from “Ca” to “Pra.” Although pattern analysis is of interest, it is not an easy approach in routine use. The calculation of the value at the end of the “Ca” period, expressed as %PPGmax, is easy to apply as routine and should facilitate V-PPG analysis in TOS-suspected patients in the future.

The sensitivity and accuracy reported for V-PPG detecting a venous positional compression in our series of recordings may appear low, but we did not expect a higher performance. Indeed, when used as a reference technique, ultrasound is the best–but probably not the ideal - candidate. First, application of the US probe to the proximal vein could have potentially interfered with venous outflow, specifically through eventual compression of collateral superficial vessels by the US probe. Second, the presence of a positional compression of the subclavian vein cannot be predictive of outflow impairment in all cases because collateral veins are able to normalize outflow despite subclavian positional compression (31). Third, it has been repeatedly reported that the sensitivity of US in detecting venous compression or occlusion is not optimal, even in trained hands and even in cases of chronic occlusion, due to bone structures making it difficult to visualize the vein at the level of compression (4). Overall, it must be kept in mind that PPG and ultrasound do not evaluate the same thing. While ultrasound detects the presence or absence of a compression, PPG evaluates the hemodynamic consequence of a compression. Then it is not surprising that the negative predictive value of PPG was high.

Issues might also result from the PPG technique itself (12, 32). First, small differences in elbow altitude relative to heart level may exist despite the recommendation to the patients to keep elbow level constant. This may result in small changes in PPG value. This fully justifies the need to determine the optimal End-Ca-PPG value and not consider values lower than 100% as abnormal. Second, extrapolation to the forearm volume from the local light absorption close to the elbow may appear to be a limitation when compared to air plethysmography (which would better account for the whole forearm volume). We believe that, on the contrary, it is an advantage for PPG. Indeed, in cases of isolated venous occlusion (without arterial occlusion) the elevated forearm will fill from the bottom to the top due to gravity and this is more likely to be rapidly recordable close to the elbow than at the wrist level. Clearly strain gauges would do the same as PPG; strain gauges have the ability to be calibrated, but they are more expensive (12, 33, 34). Furthermore, the advantage of calibrating the strain gauge recorded response compared to PPG is lost in our approach, since quantification is provided for PPG by the estimation of complete emptying during the “Pra” phase of the test. Third, and as previously discussed, PPG may remain normal despite positional occlusion of the subclavian vein due to collateral vessels. For example, it has been suggested that 2–5% of patients have a pre-clavicular vein draining blood directly to the jugular vein (35). Although this may appear to be a limitation of the PPG technique, we believe that, on the contrary, it is of major interest. Indeed, that which is clinically meaningful is forearm swelling, not venous occlusion itself. We believe that the tolerance of venous attitudinal compression depends on its hemodynamic consequences rather than the presence or absence of compression. Forth and last, the present analysis, with a value at the end of the “Ca” period, does not discriminate between outflow impairment with arterial inflow impairment and outflow impairment without arterial inflow impairment. The former should result in arrested and stable PPG during the “Ca” phase, whereas the latter should result in forearm filling from the patent artery before emptying occurs when moving in “Pra.” A low value at the end of the “Ca” period suggests that venous outflow impairment occurred but only PPG pattern classification will indicate whether arterial inflow was simultaneously present in most cases. Only a negative value at the end of the “Ca” period is indicative of a persistent arterial inflow with arrested outflow, with forearm volume exceeding the resting volume due to venous pressure exceeding the hydrostatic pressure of the resting state.

There are also some potential issues with the maneuvers that we used. First, sub-occlusion of the veins can result in slowed outflow but then result in complete emptying during the 30-s duration of the “Ca” position. We did observe this phenomenon in some patients. Analyzing the initial slope of PPG change could be proposed to account for these slowed outflows, but the speed of upper limb elevation cannot be standardized, and slow abduction would result in slow PPG changes. Another solution would have been to shorten the duration of the “Ca” period, but this would have masked the refilling that occurs because of persistent arterial inflow. On the contrary, the “Ca” period is probably too short to allow symptoms of upper limb congestion to develop. This is the reason why we did not focus on symptoms in the present study. Second, the 30-s duration is a compromise and other durations would probably result in different values at the end of the “Ca” period, but the total duration of recording of the Elcat^®^ device that we used cannot be extended above 1 min.

The absence of information on cardiac function or arrhythmia could be suggested as an issue on PPG signals. Nevertheless, the effect of arrhythmia on pulse variability would indeed interfere with pulse PPG but has little, if any, influence on VPPG. Second, the presence of cardiac failure in a population of young subjects (although we had one single patient older than 70 years old) is very unlikely.

Lastly, the fact that we did not simultaneously record PPG with ultrasound could be criticized. Nevertheless, the maneuvers for the ultrasound investigations are rarely performed simultaneously on both sides and imaging with ultrasound cannot be simultaneous on both sides.

## Conclusion and perspectives

The Ca-Pra maneuver is a simple method to allow the quantitative analysis of venous outflow with photo-plethysmography in patients with suspected TOS. This method can provide original, easy, objective and recordable proof of the presence of incomplete venous emptying during abduction and of upper limb positional swelling and could aid in the holistic approach to TOS, specifically in patients with suspected McCleery syndrome. Its test–retest reliability and sensitivity to treatments remains to be determined.

## Data availability statement

The raw data supporting the conclusions of this article will be made available by the authors, without undue reservation.

## Ethics statement

This study was performed in compliance with the principles outlined in the Declaration of Helsinki and validated by the Ethics Committee in Angers (reference: 2020/17 and accessible on Clinicaltrial.gov under reference NCT04376177). Written informed consent for participation was not required for this study in accordance with the national legislation and the institutional requirements.

## Author contributions

PA and SH performed the data analysis and are responsible for the funding and administration of the study. JH and PA wrote the draft. PR, CJ, SL, and SH criticized and revised the manuscript. All authors participated to data recording and approved the final version of the manuscript.
